# Peripheral Neuropathy Secondary to a Functional Vitamin B12 Deficiency in the Setting of Erythrocytosis

**DOI:** 10.7759/cureus.80773

**Published:** 2025-03-18

**Authors:** Liam W McDevitt, Adrian Choppa, Awais Paracha, Xinhua Zhu

**Affiliations:** 1 Internal Medicine, Northwell Health, New Hyde Park, USA; 2 Medicine-Hematology-Oncology, Northwell Health, New Hyde Park, USA

**Keywords:** erythrocytosis, functional vitamin b12 deficiency, myeloproliferative disorder, peripheral neuropathy, vitamin b12

## Abstract

Peripheral neuropathy can be a rare symptom of erythrocytosis through an ischemia-related mechanism. Vitamin B12 deficiency is another cause of peripheral neuropathy through the impaired maintenance of peripheral myelin sheaths. Patients with myeloproliferative disorders causing erythrocytosis can present clinically with functional, symptomatic vitamin B12 deficiencies despite normal serum levels. We present a case of peripheral neuropathy occurring secondary to both erythrocytosis and a functional B12 deficiency. The patient's pattern of peripheral neuropathy could not be explained by erythrocytosis alone, supporting a joint etiology involving a functional B12 deficiency. The patient was treated with therapeutic phlebotomy and vitamin B12 injections to address both underlying causes of his symptoms. He was discharged to follow up with an outpatient hematologist and reported mild symptom improvement at the two-week and one-month follow-up appointments.

## Introduction

Erythrocytosis, also known as polycythemia, is defined as an increase in total body red blood cell (RBC) mass [1]. This increase is reflected in an elevated hemoglobin or hematocrit level. Polycythemia can be divided into spurious polycythemia, caused by volume contraction, and true polycythemia, caused by an increase in RBC mass. True polycythemia can further be subdivided into primary and secondary polycythemia, depending on whether the patient has a low or elevated erythropoietin (EPO) level, respectively. The most well-known cause of primary polycythemia is polycythemia vera (PV), typically caused by a mutation in the JAK2 gene, which leads to increased signaling of the JAK-STAT pathway and clonal proliferation of erythrocyte precursors. Secondary polycythemia is often caused by conditions that would be expected to generate an increase in serum EPO levels such as smoking, hypoxia, chronic high-altitude exposure, sleep apnea, lung diseases, and EPO-secreting tumors. Elevated EPO levels can also be associated with iatrogenic causes such as anabolic steroid use or testosterone replacement therapy. Common symptoms of erythrocytosis include fatigue, headache, and dizziness. Cutaneous symptoms are less common but can include aquagenic pruritis, a ruddy facial appearance, and, rarely, peripheral neuropathy [1]. Myeloproliferative disorders causing erythrocytosis have also been observed to cause a functional vitamin B12 deficiency in patients despite normal serum levels [2]. The reason for this association is not well understood, but we believe it may be due to increased demand for vitamin B12 in the bone marrow as a consequence of uncontrolled myeloproliferation. Here, we report on a patient with a functional vitamin B12 deficiency, caused by erythrocytosis, who presented with peripheral neuropathy and lingual paresthesia. 

## Case presentation

A 64-year-old male presented to the emergency department (ED) in November 2024 with a two-week history of paresthesia in his feet. He reports that the symptoms came on gradually and described the sensation as feeling like walking on sand. Over the past week, he has also developed paresthesia in both of his hands, lips, and tongue. His lingual paresthesia has led to some reported dysphagia. 

His medical history is significant for erythrocytosis diagnosed 13 years ago. Hemoglobin at the time of diagnosis was 20.4 g/dL, and JAK2 genetic testing was negative. The patient received monthly phlebotomies with improvement in hemoglobin and hematocrit up until 2017 when he became non-compliant with follow-up visits. He eventually returned in 2020 for a follow-up, where his hemoglobin was found to be 15.2 g/dL. The patient confirmed that he did not receive any treatment related to his erythrocytosis in the interim. Past medical history is also significant for gout, which is commonly associated with erythrocytosis due to increased RBC turnover. 

The physical exam was remarkable for glossitis. Cranial nerves II-XII were intact. Strength was 5/5 in the upper and lower extremities. Range of motion was intact, and reflexes were 2+ in the upper and lower extremities as well. Sensation to light touch was preserved in the patient's lips, tongue, and extremities. His gait was normal. Laboratory workup from the ED revealed a hemoglobin of 19.6 g/dL, hematocrit of 53.2%, mean corpuscular volume (MCV) of 103.1 fL, and total bilirubin of 1.3 mg/dL. Additional testing showed an EPO level of 5.2 mU/mL, serum vitamin B12 level of 348 pg/mL, and folate level of 6.6 ng/mL, and peripheral blood was found to be negative for a JAK2 mutation by quantitative polymerase chain reaction (qPCR). Ferritin was normal, and total iron was elevated at 235 ug/dL. Electrolyte levels were all within normal ranges. The initial laboratory workup from this admission is displayed in Table 1.

**Table 1:** Initial laboratory results MCV: mean corpuscular volume; EPO: erythropoietin

Test	Value with units	Flags	Reference range
Hemoglobin	19.6 g/dL	Very high	13-17
Hematocrit	53.2%	High	39-50
MCV	103.1 fL	High	80-100
Total bilirubin	1.3 mg/dL	High	0.2-1.2
EPO	5.2 mU/mL	Normal	2.6-18.5
Serum B12	348 pg/mL	Normal	200-900
Folate	6.6 ng/mL	Normal	3.1-17.5
Total iron	235 ug/dL	High	45-165
Ferritin	148 ng/mL	Normal	30-400
Sodium	137 mmol/L	Normal	135-145
Potassium	3.8 mmol/L	Normal	3.5-5.3
Calcium	9.2 mg/dL	Normal	8.4-10.5

The patient was admitted to the hospital for therapeutic phlebotomy and further testing to determine the etiology of his neuropathy. He was tested for syphilis, human immunodeficiency virus (HIV), and lead toxicity. We checked his A1C level to rule out diabetes as a potential cause. All of these tests were negative and are displayed in Table 2. 

**Table 2:** Additional laboratory workup

Test	Value with units	Flags	Reference range
Lead blood level	2.7 ug/dL	Normal	0-3.4
*Treponema pallidum* antibody interpretation	Negative	Normal	Negative
HIV-1/2 combo result	Nonreactive	Normal	Nonreactive
A1C level	4.9%	Normal	4-5.6

In addition to a therapeutic phlebotomy, the patient also received an intramuscular injection of 1000 ug cyanocobalamin, as we considered his borderline low serum B12 levels of 348 pg/mL (200-900 pg/dL) to be a possible cause of his paresthesia. He was discharged on gabapentin for his paresthesia and referred to his original hematologist to reestablish routine care. The patient was also prescribed a weekly injection of cyanocobalamin, which he received from his hematologist. He reported mild symptom improvement at the two-week and one-month follow-up appointments with outpatient hematology but felt that the peripheral neuropathy was still bothersome. The outpatient hematologist recommended that a bone marrow biopsy may be necessary to definitively determine the cause of the patient's erythrocytosis. 

## Discussion

Despite extensive inpatient workup, the etiology of our patient's erythrocytosis remains unknown. His presentation was not consistent with volume contraction, indicating that his elevated hemoglobin and hematocrit are more likely due to true erythrocytosis. His peripheral blood was negative for a JAK2 mutation at both his initial presentation in 2011 and during his recent hospital admission. His EPO level was 5.2 mU/mL, which falls on the low end of the normal range of 4-26 mU/mL. A review of the patient's social and medical history was significant for sleep apnea and a current 40-pack-year smoking history, both of which can be contributing factors to secondary polycythemia. However, while an elevated EPO level is not required for a diagnosis of secondary polycythemia, a majority of patients tend to have EPO levels much closer to the upper limit of 26 mU/mL than our patient [3]. Despite the uncertain cause of our patient's erythrocytosis, we believe this to be the underlying cause of his peripheral neuropathy and functional B12 deficiency. 

Peripheral neuropathy classically presents as pain, numbness, tingling, or weakness following damage to peripheral nerves. It is commonly caused by systemic processes such as diabetes, aging, vitamin deficiency, autoimmune attack, or ischemia. Supported by prior case reports, peripheral neuropathy can be a rare symptom of erythrocytosis [4,5]. Patients have presented complaining of numbness and tingling in their hands and feet, similar to the paresthesia seen in our patient. The underlying mechanism of nerve damage in erythrocytosis is likely secondary to chronic ischemia, mediated by an increase in blood viscosity and platelet dysfunction [6]. An elevation in hematocrit is strongly associated with an increase in blood viscosity. Blood viscosity has an inverse relationship with flow rate and oxygen delivery; therefore, increased blood viscosity could lead to a state of chronic ischemia [7]. We believe this effect may be exacerbated in peripheral circulation as blood temperature begins to decline, fostering the precipitation of plasma proteins which further impede flow. 

While there have been reported cases of patients with erythrocytosis presenting with peripheral neuropathy, our patient is the first reported case to exhibit paresthesia around his lips and tongue in addition to his hands and feet [4,5]. We believe that a functional B12 deficiency, induced by our patient's erythrocytosis, contributed to these symptoms in addition to the chronic ischemia mechanism previously discussed. The laboratory cutoff value for a vitamin B12 deficiency is often set at 200 pg/mL, but we believe that the range of 200-400 pg/mL can be defined as the "borderline" range. This is due to the observation that patients have been diagnosed with vitamin B12 deficiencies despite having serum levels as high as 400 pg/mL, with one study estimating that up to 45% of B12-deficient patients may be missed when a serum B12 level is used in isolation [8,9]. There is also evidence to suggest that patients with myeloproliferative disorders may have a higher prevalence of B12 deficiency despite serum levels that still fall within this borderline range [2]. It is important to note that not every patient with a serum B12 level in this borderline range will have a true vitamin B12 deficiency and the sensitivity and specificity of serum B12 as a marker for deficiency vary widely depending on the study [10]. This also further highlights the importance of additional workup in determining this diagnosis. Our patient had a borderline range B12 level of 348 pg/mL, complained of lingual paresthesia despite normal electrolytes, presented with glossitis on physical exam, and was found to have an elevated MCV of 103.1 fL. These additional findings are commonly associated with a clinical vitamin B12 deficiency and support a mixed mechanism of peripheral neuropathy as a consequence of both chronic ischemia and relative vitamin deficiency secondary to untreated erythrocytosis.

Based on our patient's medical history and current presentation, erythrocytosis is the most likely cause of his functional B12 deficiency. The patient consumes meat in his diet and has no other risk factors that would support an alternative explanation for a B12 deficiency. Bone marrow requires vitamin B12 for proper erythrocytosis, and one possibility is that our patient's serum B12 levels began to decline over time in direct response to the increased demand of his bone marrow. In the setting of low serum B12 levels, we believe that the increased shunting of B12 to the bone marrow has led to a relative state of vitamin B12 deficiency elsewhere in the body. Our patient also presented with macrocytosis, which further supports that the demand for B12 in the bone marrow was even greater than what his low serum levels could provide. There have been reported cases of patients presenting with peripheral neuropathy that improved following B12 injections, despite having normal serum levels of B12 at the time of initial presentation [11]. 

The patient received weekly cyanocobalamin injections following discharge from the hospital and reported minimal symptom improvement at a two-week follow-up. This is consistent with the expected recovery timeline from a B12-related neuropathy, which has been reported to occur on the scale of weeks to months [11,12]. Complete resolution of symptoms following B12 treatment would not be expected in this patient given that his mechanism of neuropathy involves a component of chronic ischemia from underlying erythrocytosis. 

## Conclusions

Erythrocytosis can contribute to peripheral neuropathy through direct and indirect mechanisms. The most direct mechanism occurs through chronic ischemia from an increase in blood viscosity and platelet dysfunction, as previously reported in the literature. Erythrocytosis can also induce a functional vitamin B12 deficiency, thereby contributing to another cause of peripheral neuropathy through an indirect relationship. Further research is needed into the relationship between serum B12 levels and symptoms of a B12 deficiency in the setting of myeloproliferative disorders such as polycythemia, as our case highlights another example of a patient with erythrocytosis presenting with a functional B12 deficiency despite adequate serum levels. 
